# Cerebral venous thrombosis in a child with inflammatory bowel disease

**DOI:** 10.22037/ijcn.v15i3.32771

**Published:** 2021

**Authors:** Pejman ROHANI, Mohsen JAVADZADEH, Mitra KHALILI, Reyhaneh ZOJAJI

**Affiliations:** 1 Pediatric Gastroenterology, Hepatology and Nutrition Research Center, Shahid Beheshti University of Medical Sciences, Tehran,Iran.; 2 Pediatric Neurology Research Center, Shahid Beheshti University of Medical Sciences,Tehran,Iran.; 3 Pediatric Gastroenterology, Hepatology and Nutrition Research Center, Shahid Beheshti Shahid Beheshti University of Medical Sciences; 4 Pediatric Gastroenterology, Hepatology and Nutrition Research Center, Shahid Beheshti Shahid Beheshti University of Medical Sciences

**Keywords:** Children, Inflammatory bowel disease, Cerebral venous thrombosis, Case report

## Abstract

**Background:**

Inflammatory bowel disease (IBD) has both intestinal and extraintestinal manifestations. Inflammatory bowel disease is a known risk factor for cerebral venous thrombosis (CVT) in adults and children. The precise mechanism of the thrombotic event is unclear in IBD patients. We report a case of ulcerative colitis with CVT admitted for acute relapse.

**Case presentation:**

A 12-year-old boy, who was a known case of ulcerative colitis since 12 months ago, was admitted to our hospital because of bloody diarrhea and recurrent colicky abdominal pain. On the third day of admission, the patient complained of severe headache. The level of consciousness decreased gradually during 12 hours and became aphasic later. One episode of tonic-clonic seizure happened 18 hours after the onset of headache. Neurologic examination showed right hemiparesis. Physical examinations, including blood pressure and fundoscopy were unremarkable on the last admission. Brain computed tomography (CT) showed intraparenchymal hemorrhage in the left temporal lobe with asymmetric increased density in the left lateral sinus. The magnetic resonance imaging (MRI) results revealed abnormal hyperintense signal in the left lateral sinus in T1WI and T2WI, which is compatible with thrombosis (loss of signal) in magnetic resonance venography (MRV). Low-molecular-weight heparin was administered according to consultation with a hematologist and continued post-discharge. The patient’s condition improved slowly, and neurologic evaluation was normal after three months.

**Conclusion:**

Cerebrovascular events, such as cerebral venous thrombosis (CVT) or cerebral arterial infarction (CAI), are rare extraintestinal manifestations of PIBD but probably the most common forms of thromboembolism in children. Probably, treatment of CVT with anticoagulants is the best way of management. A comprehensive study is essential to understand the choice, efficacy, duration, and primary and secondary prophylaxis protocol with anticoagulants.

## Introduction

Inflammatory bowel disease (IBD) has both intestinal and extraintestinal manifestations. Intestinal symptoms include diarrhea, abdominal pain and cramping, reduced appetite and weight loss, and extraintestinal symptoms are neurological, dermatological, psychological, and thrombotic disorders. Inflammatory bowel disease is a known risk factor for cerebral venous thrombosis (CVT) event in adults with the prevalence of 1.3-6.4%. (1-5) In children, the prevalence of CVT events is estimated to be 3.3% during the course of the disease (1, 6). The precise mechanism of the thrombotic event in patients with IBD is not known. It seems that disease exacerbation, severe pancolitis, prolonged hospitalization, indwelling catheters, and immobilizations are some of the important predisposing factors. We report a case of ulcerative colitis with CVT during admission for acute relapse. 

## Case Presentation

A 12-year-old boy, who was a known case of ulcerative colitis since 12 months ago, was admitted to our hospital because of bloody diarrhea and recurrent colicky abdominal pain. On the third day of admission, the patient complained of severe headache. The level of consciousness decreased gradually during 12 hours, and he became aphasic later. One episode of tonic-clonic seizure happened 18 hours after the onset of the headache. Neurologic examination showed right hemiparesis. The patient had had three episodes of flare during 12 months since diagnosis and was responsive to methylprednisolone and was corticosteroid-dependent. His physician had started azathioprine in the last relapse. During treatment with azathioprine, the patient became neutropenic, and azathioprine was stopped by the parents but without any substitute. The parents had not contacted their own physician until the last relapse. Physical examinations, including blood pressure and fundoscopy, were unremarkable at the last admission time. The lab studies are summarized in Table 1.

**Figure1:**
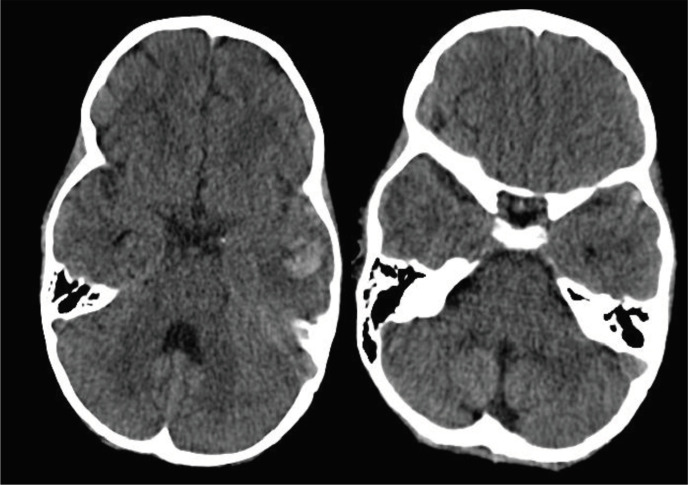
Brain CT: Intraparenchymal hemorrhage in the left temporal lobe with asymmetric increased density in the left lateral sinus

Brain CT showed intraparenchymal hemorrhage in the left temporal lobe with asymmetric increased density in the left lateral sinus (Fig. 1). The MRI results showed an abnormal hyperintense signal in the left lateral sinus in T1WI and T2WI, which is compatible with thrombosis (loss of signal) in magnetic resonance venography (MRV) (Fig 2 A, B). 

The child was admitted to PICU under supportive care. Treatment of the ulcerative colitis (UC) flare was performed, and low-molecular-weight heparin was administered according to consultation with a hematologist and continued post discharge. The child’s condition improved slowly, and neurologic evaluation was normal after three months. Now, UC is in remission, and the patient is being treated with infliximab.

## Discussion

Adults and children with IBD are susceptible to systemic thromboembolism (TE). There are different kinds of thromboembolic events as pulmonary embolism, deep venous thrombosis, thrombophlebitis, portal vein thrombosis, Budd-Chiari syndrome and intracranial venous sinus thrombosis (7). The incidence of TE in children with IBD is lower than in adults (8). Cerebrovacular events, such as cerebral venous thrombosis (CVT) or cerebral arterial infarction (CAI), are rare extraintestinal manifestations of PIBD, but they are probably the most common forms of thromboembolism in children (9). Cerebral venous thrombosis is probably secondary to coagulation disorders, such as increased platelet activation, the activation of the coagulation cascade, and impaired fibrinolysis (1, 10). Thrombosis in IBD cases may also happen due to endothelial dysfunction in the regulation of inflammation, coagulation, and vascular repair (11). 

 There are lots of risk factors, including thrombocytosis (12), increased procoagulation state (13-15), decreased anticoagulation state (16-22), and hyperhomocysteinemia (23-25). The well-known risk factors for TE like indwelling catheters, surgery, infection, prolonged hospitalization, dehydration, and immobilization predispose children with IBD to cerebrovascular events. The most important of them are summarized in Table 2. There are different opinions about the effect of therapy with corticosteroids as a risk factor for TE (9). In addition, it is not known whether biologic therapy as a new treatment modality in children may prevent or predispose patients to TE (26, 27). 

We do not know what kinds of patients are more prone to CVT. Many cases in the extant literature had the active disease (9), while in some of them, TE happened at disease onset (28, 29). But more rarely in some patients, the disease was in remission or even many years after colectomy (30-32). According to Lazzerini et al., early diagnosis, correct treatment, and evaluation of patients for risk factors of TE are some of the important steps to prevent thrombosis (9). The best way to manage children for secondary, the chance of recurrence and patients who are at risk are not recognized. 

Probably, CVT treatment with anticoagulants is the best management strategy, although there are case reports without anticoagulant treatment (33). A comprehensive study is essential to understand the choice, efficacy, duration, and primary and secondary prophylaxis protocol with anticoagulants. 

**Figure2 A:**
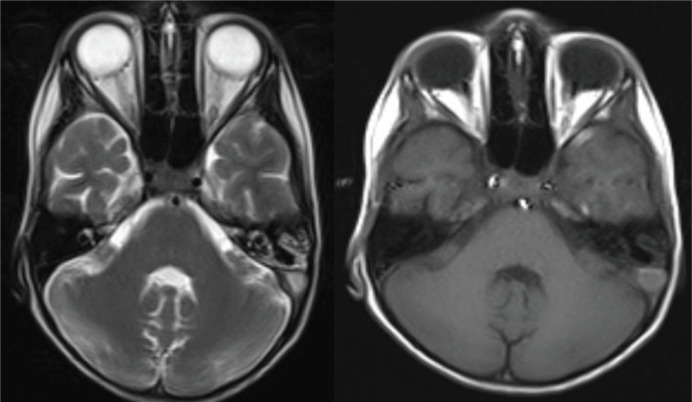
Brain MRI: Abnormal hyperintense signal in left lateral sinus in T1WI & T2WI

**Figure2 B:**
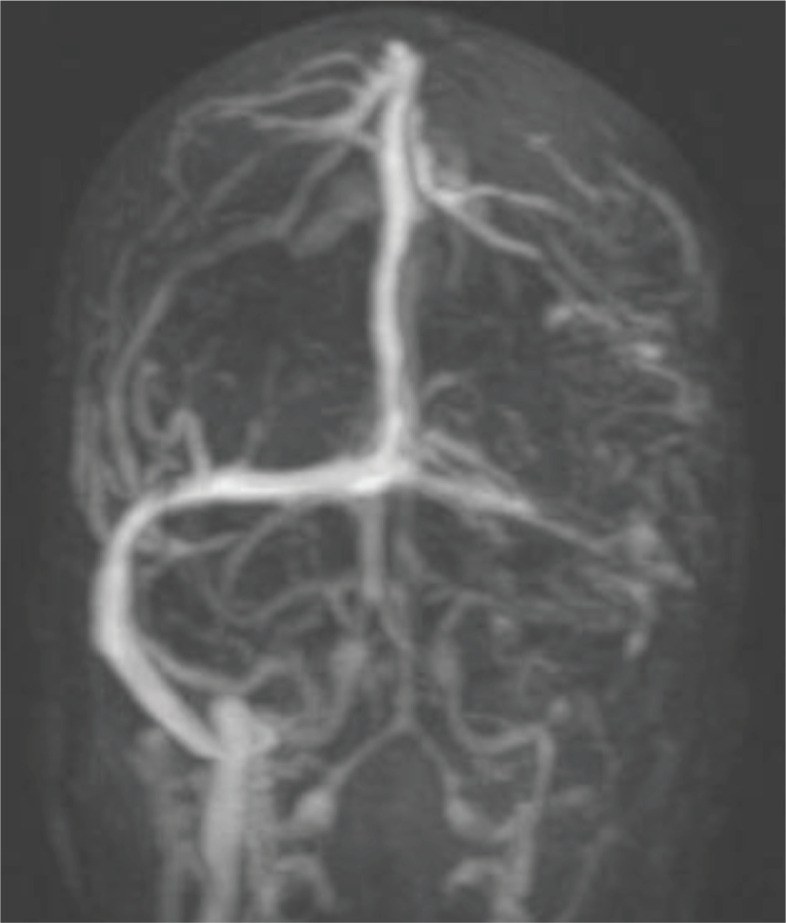
Brain magnetic resonance venography (MRV): Abnormal hyperintense signal in the left lateral sinus in T1WI and T2WI is compatible with thrombosis (loss of signal) in MRV

**Table 1:** Lab data

**Test**		Result	Normal
**Factor VII**		65	58-115
**Factor VIII**		109 %	53-131
**Factor XI**		100	50-97
**Protein C**		106	55-111
**Protein S**		79	52-92
**Anti Thrombin **		143 %	80-120
**Homocysteine (plasma) **		10 micmol/L	5-15
**Prothrombin G2021OA PCR**		Wild type Homozygous	
**MTHFR 677 PCR**		Wild type Homozygous	
**MTHFR 1298 PCR**		Heterozygous mutation	
**Factor V Leiden**		Wild type Homozygous	
**Phospholipid Ab IgG**		5.7 U/ml	<12
**Phospholipid Ab IgM**		4.7 U/ml	<12
**Cardiolipin Ab IgG**		2.2 U/ml	<12
**Cardiolipin Ab IgM**		4.6 U/ml	<12
**Lupus Anticoagulant**		30	25-40
**PANCA**		1.9	<12=Negative
**CANCA**		156	<12=Negative
**Anti ds DNA**		47	Normal<16
**C3**		123	90-180
**C4**		22	10-40
**CH50**		55	51-150
**FANA**		Negative	Negative<1/80
**HLA-B5**		Negative	
**HLA-B51**		Negative	
**Betta Glycoprotein**		0.5	Normal<16

**Table 2:** Risk factors of thromboembolism in children with IBD

**Increased procoagulation **	**General risk factors**
Factor V Leiden	Disease flare
MTHFR mutations	Immobilization
Antiphospholipid antibodies	Indwelling catheters
Hyperhomocysteinemia	Dehydration
Thrombocytosis	Hypertension
**Decreased anticoagulation**	Obesity
Protein c deficiency	Oral contraceptive
Protein s deficiency	Infection
Prothrombin gene mutation	Surgery
**Family history of thromboembolism**	Prolong hospitalization
